# Prevalence and Incidence of Latent Tuberculosis Infection in Georgian Healthcare Workers

**DOI:** 10.1371/journal.pone.0058202

**Published:** 2013-03-25

**Authors:** Jennifer A. Whitaker, Veriko Mirtskhulava, Maia Kipiani, Drew A. Harris, Nino Tabagari, Russell R. Kempker, Henry M. Blumberg

**Affiliations:** 1 Division of Infectious Diseases, Emory University School of Medicine, Atlanta, Georgia, United States of America; 2 National Center for Tuberculosis and Lung Diseases, Tbilisi, Georgia; 3 Department of Internal Medicine, Emory University School of Medicine, Atlanta, Georgia, United States of America; 4 "AIETI" Medical School, David Tvildiani Medical University, Tbilisi, Georgia; 5 Divisions of General Internal Medicine and Infectious Diseases, Mayo Clinic, Rochester, Minnesota, United States of America; Copenhagen University Hospital, Denmark

## Abstract

**Background:**

Tuberculosis is a major occupational hazard in low and middle-income countries. Limited data exist on serial testing of healthcare workers (HCWs) with interferon-γ release assays (IGRAs) for latent tuberculosis infection (LTBI), especially in low and middle-income countries. We sought to evaluate the rates of and risk factors for LTBI prevalence and LTBI test conversion among HCWs using the tuberculin skin test (TST) and QuantiFERON-TB Gold In-tube assay (QFT-GIT).

**Methods:**

A prospective longitudinal study was conducted among HCWs in the country of Georgia. Subjects completed a questionnaire, and TST and QFT-GIT tests were performed. LTBI testing was repeated 6-26 months after baseline testing.

**Results:**

Among 319 HCWs enrolled, 89% reported prior BCG vaccination, and 60% worked in TB healthcare facilities (HCFs). HCWs from TB HCFs had higher prevalence of positive QFT-GIT and TST than those from non-TB HCFs: 107/194 (55%) vs. 30/125 (31%) QFT-GIT positive (p<0.0001) and 128/189 (69%) vs. 64/119 (54%) TST positive (p = 0.01). There was fair agreement between TST and QFT-GIT (kappa = 0.42, 95% CI 0.31–0.52). In multivariate analysis, frequent contact with TB patients was associated with increased risk of positive QFT-GIT (aOR 3.04, 95% CI 1.79–5.14) but not positive TST. Increasing age was associated with increased risk of positive QFT-GIT (aOR 1.05, 95% CI 1.01–1.09) and TST (aOR 1.05, 95% CI 1.01–1.10). High rates of HCW conversion were seen: the QFT-GIT conversion rate was 22.8/100 person-years, and TST conversion rate was 17.1/100 person-years. In multivariate analysis, female HCWs had decreased risk of TST conversion (aOR 0.05, 95% CI 0.01–0.43), and older HCWs had increased risk of QFT-GIT conversion (aOR 1.07 per year, 95% CI 1.01–1.13).

**Conclusion:**

LTBI prevalence and LTBI test conversion rates were high among Georgian HCWs, especially among those working at TB HCFs. These data highlight the need for increased implementation of TB infection control measures.

## Introduction

Tuberculosis (TB) is a significant occupational health hazard for healthcare workers (HCWs). TB transmission in healthcare facilities can be significantly reduced with the implementation of effective TB infection control measures [1]–[4]. The nosocomial transmission of multi-drug resistant (MDR-TB) and extensively drug resistant TB (XDR-TB) further highlights the need for effective TB infection control measures [5]–[7]. While most high-income countries have successfully implemented TB infection control measures [2], TB infection control measures are limited or virtually non-existent in most resource-limited countries [8], [9].

Most high-income countries screen HCWs periodically for latent tuberculosis infection (LTBI) as part of their TB infection control programs [2], [10] but this practice is unusual in most low and middle-income countries. For many years the tuberculin skin test (TST) was the only test available for diagnosis of LTBI; however, the interferon-γ release assays (IGRAs), T-cell based assays, have recently become available and provide alternative diagnostic test for LTBI [11]. Two commercially available IGRAs have been approved for use by the U.S. FDA—the QuantiFERON-TB Gold In-Tube (QFT-GIT) assay (Cellestis Inc., Valencia CA) and the T-SPOT.TB assay (Oxford Immunotec, Abingdon, UK). IGRAs have several advantages over the TST: they require only one visit, are not affected by BCG vaccination, have less cross-reaction with non-tuberculous mycobacteria, are less subjective in measuring results, and can be repeated without boosting. However, there is a lack of data on how IGRAs perform when used for serial testing, especially in low and middle-income countries.

In 2005, the U.S. Centers for Disease Control (CDC) recommended that IGRAs can be used in all settings where the TST has been used, including the serial testing of healthcare workers [12]. The updated 2010 CDC guidelines caution that “lenient criterion to define IGRA conversion might produce more conversions than are observed with the more stringent criteria applied to TSTs. Furthermore, an association between an IGRA conversion and subsequent disease risk has not been demonstrated. The new criteria for interpreting changes in an IGRA that identify new infections remain uncertain” [11]. Guidelines from Australia advise caution when using IGRAs for HCW screening [13], and Canadian guidelines do not recommend the use of IGRAs for serial testing of HCWs [14], citing a lack of available data. A World Health Organization (WHO) policy statement on the use of IGRAs in low- and middle-income countries indicates that “data on serial testing and reproducibility of IGRAs, as well as evidence on the predictive value of IGRAs in health care workers (HCWs), are still absent for high-incidence settings" [15].

The country of Georgia has high rates of tuberculosis. In 2010, the incidence of TB in Georgia was 107 cases/100,000 population, and prevalence was 118 cases/100,000[16]. Emory University has a longstanding collaboration with institutions in the country of Georgia in the health, educational, research, and scientific sectors that spans more than 20 years following the break-up of the Soviet Union. For over a decade, the Emory Division of Infectious Diseases has had a long standing collaboration with the Georgian National TB Program and National Center of TB and Lung Diseases (NCTLD). Physicians from both countries agreed that screening Georgian HCWs for LTBI and evaluating LTBI test conversion rates provides important information on TB occupational exposure risks for Georgian HCWs. In addition, the use of both TST and QFT-GIT LTBI tests allows for the comparison of the two tests. As there is limited data on QFT-GIT in serial testing, we thought that our study would contribute to the literature in this area. In addition, through long term follow up of this cohort, we will be able to provide data regarding the association between IGRA conversion and risk of developing active TB disease.

The purpose of our study was to determine the prevalence and risk factors for LTBI among HCWs in the country of Georgia and to assess rates and risk factors for LTBI test conversion. We performed a prospective longitudinal study of HCWs in TB and non-TB facilities in Georgia using serial testing with the TST and the QFT-GIT. We also sought to determine the effect of occupational exposure to TB on the outcome of TST and QFT-GIT positivity at baseline and conversion of these tests.

## Methods

### Study Setting and Population

A prospective longitudinal study was conducted from 2009–2011. HCWs from the Georgian National TB Program (NTP), including the National Center for Tuberculosis and Lung Diseases (NCTLD) in Tbilisi, its affiliated outpatient clinics, as well as HCWs from non-TB facilities were eligible to enroll. Inclusion criteria were age ≥18 years old, HCW in the country of Georgia, and provision of written informed consent. Exclusion criteria were history of active TB and type I hypersensitivity reaction to the purified protein derivative used in the TST. A convenience sampling method was used. This was a voluntary study. HCWs were approached with information about the study at their place of work and were enrolled if they agreed to participate and provided informed consent. HCWs completed a questionnaire with demographic information, medical history, and employment history. BCG vaccination status was assessed by self-reporting and by visual inspection for a BCG scar.

HCWs who tested positive for LTBI by either test were referred to the NCTLD for evaluation to rule out active TB. This evaluation included chest x-ray and symptoms screening. As it is not the standard of care in Georgia, no HCWs were treated for latent TB infection.

### Ethics Statement

The study was approved by the Emory University Institutional Review Board and Georgian NCTLD Ethics Committee. HCWs enrolled into the study provided written informed consent in their native Georgian language of Kartuli.

### TST and QFT-GIT Assay

After completing the questionnaire, two diagnostic tests for LTBI were performed. Three ml of blood was drawn for the QFT-GIT test, which was performed according to the manufacturer's instructions and as previously described [17]. IFN-γ values >10 IU/ml were treated as 10 IU/ml. Repeat QFT-GIT testing was performed on participants 6–26 months after baseline testing. QFT-GIT was performed on all participants who underwent repeat testing. As recommended by the manufacturer and the CDC [11], the QFT-GIT result was defined as positive if the response to the TB antigens minus the negative control was ≥0.35 IU/ml and >25% of the negative control, negative if these criteria were not met, and indeterminate if either the negative control had a result of >8 IU/ml or if the positive control had a result of <0.5 IU/ml. According to the American Thoracic Society (ATS) and CDC guidelines, the TST was defined as positive if the induration in HCWs was ≥10 mm [18], [19]. According to CDC guidelines, a QFT-GIT conversion was defined as a baseline interferon-gamma (IFN-γ) <0.35 IU/ml and a follow-up IFN-γ level ≥0.35 IU/ml, without any consideration of the magnitude in change of the IFN-γ response [11]. A QFT-GIT reversion was defined as a baseline IFN- γ ≥0.35 IU/ml and a follow-up IFN-γ level <0.35 IU/ml. The TST was performed using the Mantoux method [22] and read 48–72 hours after placement. The TST was placed using 5 tuberculin units (TU) of PPD (Tubersol®, Connaught; Swiftwater, PA, USA). According to ATS and CDC guidelines, a TST conversion for a healthcare worker was defined as a change in induration from <10 mm to ≥10 mm, with an increase of ≥10 mm within 2 years [11], [18]. Only patients with a negative baseline TST had repeat TST testing performed at follow up.

Repeat testing was performed over a range of 6–26 months due to limited research study staff and inability to test large numbers of healthcare workers at the same time. Due to limited research study staff and limited resources, not all HCWs were offered repeat testing. Repeat testing was performed by convenience sampling.

### Statistical Analysis

Data were collected and managed using a REDCap electronic data capture tool. REDCap (Research Electronic Data Capture) is a secure, web-based application designed to support data capture for research studies [20]. Statistical analyses were performed using SAS software version 9.2 (SAS Institute, Inc., Cary, NC, USA). For determination of prevalence of positive TST, we included participants who had TST performed in our study or reported prior history of positive TST (n = 308). For determination of prevalence of positive QFT-GIT, we included participants who had QFT-GIT measured (n = 319). Concordance between the two diagnostic tests for LTBI (TST and QFT-GIT) was determined using the kappa (κ), where κ>0.75 represents excellent agreement, κ = 0.4–0.75 represents fair to good agreement, and κ<0.4 represents poor agreement [21]. Only data from participants who had measured TST and QFT-GIT values at baseline (n = 260) was included for the concordance analysis. Occupational TB exposure frequency was categorized as daily (contact to TB patients ≥ 5 days per week), frequent (contact<5 days per week and ≥ twice per month), rare (contact < twice per month and ≥ once per 3 months), and very rare (contact < once per 3 months). The occupational TB exposure variable was later dichotomized into frequent (defined as contact ≥ twice per month) and rare (defined as contact < twice per month) for univariate and multivariate logistic regression analysis.

Multivariate analysis was performed using logistic regression modeling with outcomes of TST positivity and QFT-GIT positivity. Participants were included in these models if they had measured TST (or documented history of positive TST) and measured QFT-GIT. The same participants were included in the models for QFT-GIT positive and TST positive. Variables included in the final multivariate models were chosen on the basis of biologic plausibility of their association with the outcomes, in addition to the statistical and epidemiological criteria. A p-value ≤0.05 was defined as statistically significant. Interaction terms were created based on biologic plausibility and were tested individually for significance with the Likelihood Ratio Test [22]. Incidence rates for TST and QFT-GIT conversion (in 100 person-years) were determined by dividing the number of events by the total amount of person-time contributed by those who were negative at time of first testing and accounting for the time to follow-up testing. Risk factors for TST and QFT-GIT conversion were determined by univariate logistic regression analysis and multivariate logistic regression analysis.

## Results

### The Study Population

**Figure 1:**
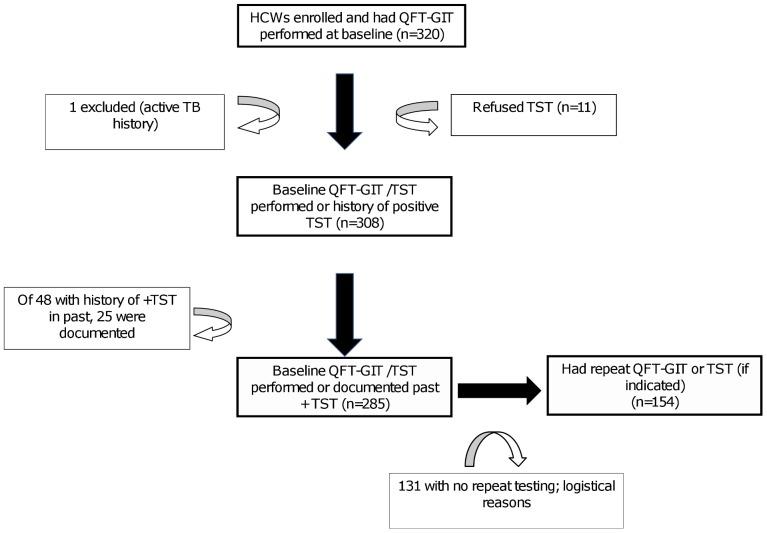
Enrollment and follow-up of participants. HCW: healthcare workers, QFT-GIT  =  QuantiFERON-TB Gold In-tube assay, TST = tuberculin skin test.

Between March 2009 and July 2011, 320 Georgian health care providers were enrolled in the study (Figure 1); all enrolled had a QFT-GIT performed. One participant was excluded for history of active TB disease. Fifty-nine HCWs did not have a TST performed (48 participants reported a prior positive TST in the past and 11 refused to have a TST done). We were able to confirm documentation of a positive TST for 25 of the 48 HCWs with a prior history of testing positive.

The characteristics of the study population (n = 319) are described in Table 1. The majority of HCWs in our study were from Tbilisi (n = 274, 86%), the capital of Georgia where the NCTLD is located. More than 60% (194/319) of the participants worked in TB facilities, and nearly 40% worked in non-TB facilities. The majority of the participants were female (n = 259, 81%). The mean age was 40.9 years (standard deviation 12.6 years). The mean number of years in healthcare was 17.0. Forty nine percent of the HCWs reported frequent TB exposure at work (contact ≥ twice per month), and 51% reported rare TB exposure at work (contact ≤ once a month).

**Table 1:** Demographic information for healthcare workers (n = 319).

Variable	Subcategory	*n* (%)
Age, years	18–29	64 (20%)
	30–39	83 (26%)
	40–49	98 (31%)
	≥50	74 (23%)
Gender	Female	259 (81%)
	Male	60 (19%)
Georgian ethnicity		305 (96%)
Location	Tbilisi	274 (86%)
	Other	45 (14%)
Positive BCG vaccination history		285 (89%)
Healthcare facility	TB inpatient facility	122 (38%)
	TB outpatient facility	72 (23%)
	Non-TB health facility	70 (22%)
	Medical school	11 (3%)
	Other	44 (14%)
Years in healthcare	0–4	70 (22%)
	5–14	83 (26%)
	15–24	72 (23%)
	>25	94 (29%)
Occupation	Administrative staff	94 (29%)
	Medical students	14 (4%)
	Nurses	50 (16%)
	Physicians	114 (36%)
	Other	47 (15%)
Education	Graduate school	230 (72%)
	Undergraduate	68 (21%)
	Secondary school or less	21 (4%)
TB exposure frequency	Daily	102 (32%)
	Frequent(<5 days/week and ≥ twice a month)	56 (17%)
	Rare (≤ once a month and ≥ once a quarter)	60 (19%)
	Very rare (< once a quarter)	101 (32%)
Positive history of TB contact in home		20 (6%)

### Prevalence of TST and QFT-GIT Positivity and Concordance

The prevalence of a positive TST at baseline was 67% (193/308), and the prevalence of a positive QFT-GIT was 46% (146/319). Among HCWs who worked in TB facilities, 107 of 194 (55%) had a positive QFT-GIT vs. 30 of 125 (31%) of HCWs working in non-TB facilities (p<0.0001). Among HCWs working in TB-facilities, 128 of 189 (69%) had positive TST vs. 64 of 119 (54%) of those working in non-TB facilities (p = 0.01).

Among HCWs who had both tests measured (n = 260), 64% had at least one positive LTBI test. There was fair concordance between the TST and QFT-GIT [κ]  = 0.42 [95% CI: 0.31–0.52]). Agreement between the two diagnostic tests for LTBI was 70.4%; with 36% (93/260) of tests concordantly negative, 35% (90/260) tests concordantly positive, 21% (55/260) TST positive and QFT-GIT negative, and 8% (22/260) QFT-GIT positive and TST negative. Concordance between the two LTBI tests was higher among those HCWs with no history of BCG vaccine (n = 27), κ = 0.70 (95% CI: 0.44–0.97) than the group with a BCG vaccine history (n = 233), κ = 0.38 (95% CI: 0.27–0.50).

### Risk factors for LTBI prevalence

In multivariate analysis, independent risk factors for positive TST included age in years (aOR 1.05, 95% CI 1.01–1.10) and occupation as a nurse compared to occupation as administrative staff (aOR 2.77, 95% CI 1.01–7.55) (Table 2). In multivariate analysis, independent risk factors for positive QFT-GIT included frequent contact with TB patients (aOR 3.04; 95% CI 1.79–5.14) and age in years (aOR 1.05, 95% CI 1.01–1.09) (Table 2).

**Table 2:** Multivariate analysis for risk factors for a positive TST and QFT-GIT among Georgian healthcare workers (n = 285).

Variable	Positive TST	Positive QFT-GIT
	Adjusted OR	Adjusted OR
	(95% CI)	(95% CI)
Frequent vs. rare contact with TB patients	1.29 (0.76–2.18)	3.04 (1.79–5.14)
Gender (F vs. M)	1.11 (0.53–2.35)	0.55 (0.25–1.20)
Age in years	1.05 (1.01–1.10)	1.05 (1.01–1.09)
Years in healthcare (continuous, in years)	0.99 (0.95–1.03)	0.99 (0.96–1.04)
BCG vaccine history (positive vs. negative)	1.16 (0.51–2.63)	0.84 (0.36–1.94)
Occupation		
Med Stud vs. Admin	0.58 (0.15–2.23)	1.25 (0.28–5.57)
Nurse vs. Admin	2.77 (1.01–7.55)	1.75 (0.70–4.32)
Physician vs. Admin	0.42 (0.19–0.95)	0.60 (0.24–1.52)
Other vs. Admin	0.67 (0.27–1.64)	0.80 (0.35–1.82)

TST  =  Tuberculin skin test

QFT-GIT  =  QuantiFERON-TB Gold In-tube assay

Frequent  =  contact with TB patients ≥ twice per month

Rare  =  contact with TB patients < twice per month

Admin  =  Administrative/technical staff (reference group)

### TST and QFT-GIT conversion rates and QFT-GIT reversions

**Figure 2:**
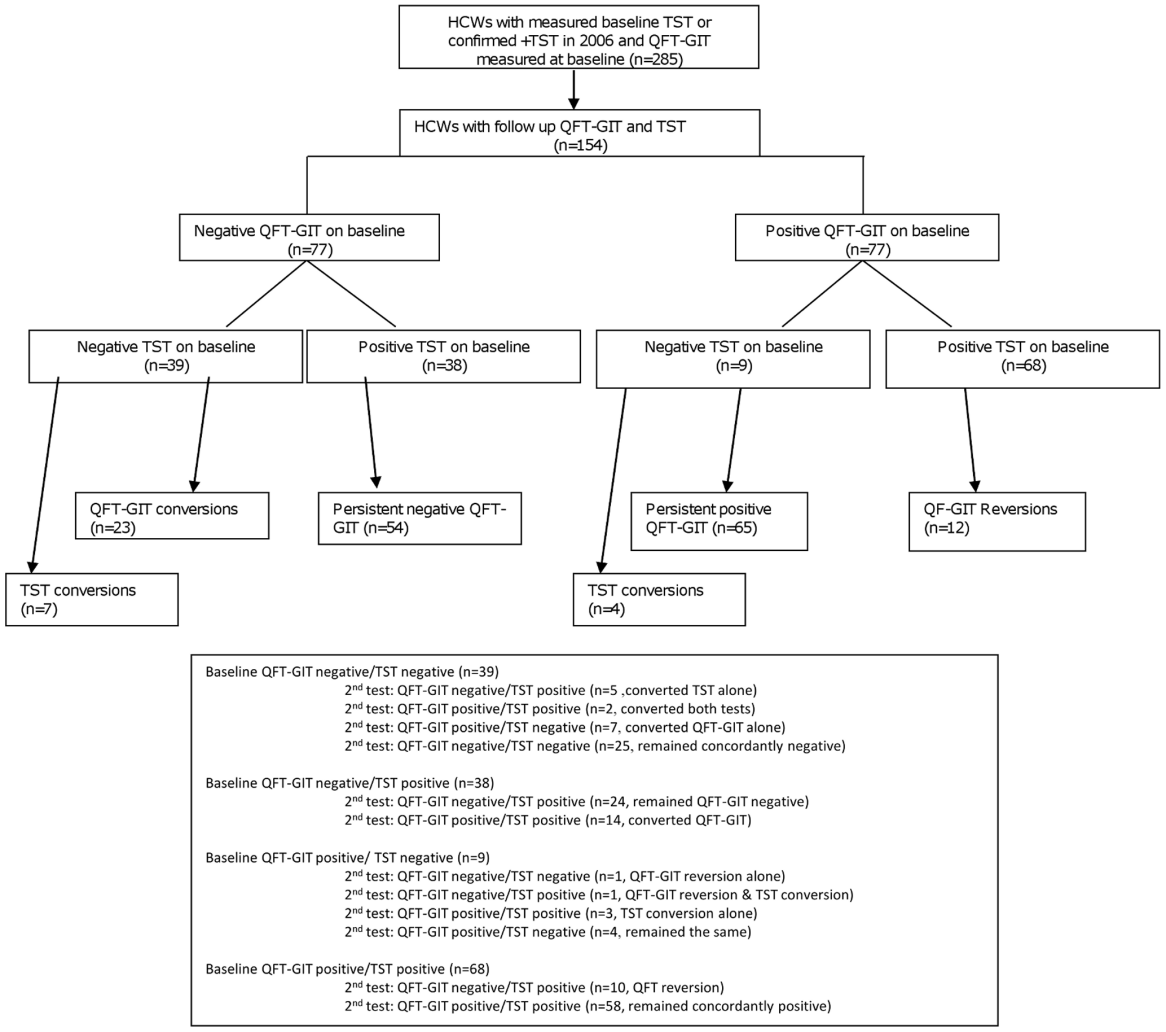
Results of Diagnostic tests for Latent TB Infection among Healthcare Workers (HCWs) who underwent serial testing: Conversions and Reversions. HCW: healthcare workers, QFT-GIT  =  QuantiFERON-TB Gold In-tube assay, TST  =  tuberculin skin test.

Among the 154 HCWs who had TST and QFT-GIT performed at baseline and had repeat or serial testing, 77 (50%) were susceptible to QFT-GIT conversion (negative QFT-GIT at baseline) and 48 (31%) were susceptible to TST conversion (negative TST at baseline) (Figure 2). The median time from baseline to repeat LTBI testing was 16 months (range 6–26 months). QFT-GIT conversions were documented among 23 (29.9%) of 77 HCWs. TST conversions occurred in 11 (22.9%) of 48 HCWs (Figure 2). The conversion rate by QFT-GIT regardless of baseline TST result was 22.8/100 person-years. The conversion rate by TST regardless of baseline QFT-GIT result was 17.1/100 person-years. The conversion rate by either test among those who had concordantly negative TST and QFT-GIT results at baseline was 26.9/100 person-years (17.3/100 person-years for TST conversion and 13.5/100 person-years for QFT-GIT conversion).

Among the 77 HCWs who had a positive QFT-GIT at baseline testing, 12 had QFT-GIT reversions for an overall QFT-GIT reversion rate of 15.6% and a reversion rate of 13.4/100 person-years.

### Risk factors for TST and QFT-GIT conversion

In univariate analysis, female HCWs had a lower risk of TST conversion (OR = 0.13, 95% CI 0.03–0.59) and HCWs who worked at TB facilities were at increased risk for a TST conversion (p = 0.04). All of the TST conversions occurred among HCWs who worked in TB healthcare facilities.

In multivariate analysis, being a female HCW was associated with decreased risk of TST conversion (OR 0.05, 95% CI 0.01–0.43) (Table 3).

**Table 3:** Multivariate analysis for latent tuberculosis infection (LTBI) diagnostic test (QFT-GIT and TST) conversion.

	QFT-GIT conversion (regardless of TST)	TST conversion (regardless of QFT-GIT)	Convert either test from TST-/QFT-GIT-
	(23/77)	(11/48)	(14/39)
	Adjusted OR (95% CI)	Adjusted OR (95% CI)	Adjusted OR (95% CI)
Frequent vs. rare contact with TB patients	1.09 (0.38–3.13)	4.29 (0.63–29.06)	5.61 (0.90–34.8)
Gender (F vs. M)	0.67 (0.17–2.68)	0.05 (0.01–0.43)	0.07(0.01–0.63)
Age in years	1.07 (1.01–1.13)	1.05 (0.96–1.14)	1.08 (0.99–1.16)

Frequent contact is contact with TB patients ≥ twice per month

Rare contact is contact with TB patients < twice per month.

In univariate analysis, increasing age per year (OR 1.06 per year, 95% CI 1.01–1.12) was associated with increased risk of QFT-GIT conversion. Age in years was also associated with increased risk of QFT-GIT conversion in multivariate analysis (OR 1.07 per year, 95% CI 1.01–1.13). In multivariate analysis of risk of conversion of either test (QFT-GIT or TST), only female gender was found to be a significant independent risk factor, and it was negatively associated with risk of LTBI test conversion (OR 0.07, 95% CI 0.01–0.63) (Table 3).

### Active TB Disease

Only one HCW was diagnosed with active TB disease after symptom screen and chest x-ray at time of LTBI testing. Three HCWs did develop active disease during the course of this study. These HCWs had tested positive both by TST and QFT within 12 months before being diagnosed with active TB. It is expected that TB cases are under-reported among HCWs to the NCTLD/NTP TB surveillance department due to the stigma associated with having TB disease. There is no access available to TB diagnosis and treatment without notification to the NCTBLD/NTP TB surveillance department.

## Discussion

We found a high prevalence of LTBI among Georgian healthcare workers, which was significantly higher among HCWs at TB facilities (55% QFT-GIT positive and 69% TST positive) compared to HCWs at non-TB healthcare facilities (31% QFT-GIT positive and 54% TST positive). Additionally, alarmingly high rates of LTBI diagnostic test conversions were found among Georgian HCWs. All 11 TST conversions and 18/23 (78%) QFT-GIT conversions occurred among HCWs working at a TB facility. These findings highlight a high rate of ongoing transmission of TB in Georgian healthcare facilities (HCFs) especially TB HCFs, and the urgent need to implement effective TB infection control measures.

There are limited data on the serial testing of HCWs using IGRAs. Furthermore, the dearth of data is most pronounced in low and middle-income countries where the prevalence of TB is the highest. Our study provides valuable data on serial IGRA testing in a highly endemic TB country. To our knowledge, there is only one published study of serial testing of HCWs using IGRAs in a high burden TB country (India) [23]. Three cross-sectional studies have evaluated IGRA performance in high burden TB countries: India [24], Russia [25], and Vietnam [26]. Among these cross-sectional studies of HCWs in high burden TB countries, TST and QFT-GIT positivity rates were high (40–66%) [23]–[27]. Although Georgia is not designated by WHO as one of the 22 high burden TB countries based on absolute number of TB cases [28], it does have a high incidence and prevalence of TB (higher than some of the WHO designated high burden TB countries) and has been designated by the WHO as a high burden MDR-TB country [16]. It is particularly important to assess the risk of nosocomial transmission of TB to HCWs in the setting of a highly endemic M/XDR-TB country, as there are no evidence based guidelines for treatment of LTBI due to M/XDR TB contact [29].

The most striking findings of our study were the high rates of LTBI test conversion representing probable recent infection with *M. tuberculosis* among HCWs. In our study the QFT-GIT conversion rate was 22.8/100 person-years, 17.1/100 person-years for TST, and 26.9/100 person-years for either test conversion among those who had both negative TST and QFT-GIT results at baseline. To put these rates into perspective, a study of HCWs in India found TST conversion rates of 2.7/100 person-years and QFT-GIT conversion rates of 7.7/100 person-years [23]. A study of Malaysian healthcare workers found QFT-GIT conversion rates of 9.9/100 person-years [30]. Of note, these other studies were of HCWs from hospitals not specializing in TB care, whereas in our study, 60% of HCWs worked in facilities specializing in TB care. Nonetheless, our findings support the need for strengthening of TB infection control measures in Georgian healthcare facilities, particularly those specializing in TB care.

Our study is the first study of serial IGRA testing in a highly endemic TB country that also evaluates the relationship between degree of exposure to patients with active tuberculosis and risk of LTBI test conversion. The one published study of serial testing of HCWs using IGRAs did not evaluate the relationship between degree of TB exposure and LTBI test conversion [23]. In multivariate analysis, frequent contact with TB patients (contact ≥ twice per month) was associated with at least one LTBI test being positive (OR 1.77, 95% CI 1.03–3.05) and also with a positive QFT-GIT (OR 3.04, 95% CI 1.79–5.14). This association was not seen from our prior LTBI study in 2006 of Georgian HCWs [27], likely as a result of 81% of the HCWs working in TB facilities, and thus few HCWs without frequent TB contact to compare with. Other studies have found a positive association between occupational TB exposure and IGRA positivity rates [31]. Of three cross-sectional studies of IGRAs and TST conducted in high-incidence settings [24]–[26], only one study from India evaluated the association between occupational risk factors for both TST and IGRA [24]. This study found a stronger, but non-significant, association between occupational risk factors and IGRA positivity than for TST positivity [24]. In the systematic review by Zwerling et al., among 22 cross-sectional studies of HCWs in low and moderate incidence TB countries, TST, QFT-GIT, and TSPOT.TB correlated well with established indicators of occupational risk of TB exposure, although no test was more consistently associated with these indicators of exposure [31].

Our study was not designed to evaluate whether QFT-GIT or TST is superior for serial LTBI testing, but rather to assess for risk factors associated with each test conversion. While we did not observe a significant association with TB exposure frequency and TST or QFT-GIT test conversion, we did find that the majority of TST (100%) and QFT-GIT (78%) conversions occurred in TB facilities. Our small sample size for serial testing: only 48 HCWs were TST negative on baseline testing and only 77 were negative on baseline QFT-GIT testing, limited our ability to detect significance. A study from Japan of serial testing of HCWs with QFT-GIT found that HCWs who worked in a TB ward were 20 times more likely to experience QFT-GIT conversion than those who did not work in a TB ward. This Japanese study did not perform serial TST tests, so no comparison could be made with TST conversions [32].

Interestingly, we observed in multivariate analysis, that females had decreased risk of LTBI test conversion for either test (aOR 0.07, 95% CI 0.01–0.63). When each test conversion was analyzed separately in multivariate analysis, females had decreased risk for TST conversion (aOR 0.05, 95% CI 0.01–0.43) but not for QFT conversion (aOR 0.67, 95% CI 0.17–2.68). During the course of this study, respirators at TB facilities in Tbilisi were introduced. One possible explanation for the decreased LTBI test conversion rates among females is that female HCWs were more likely to wear and comply with respirator use while in contact with known TB patients than male HCWs. However, since we did not collect data on HCW use of respirators, we cannot make this conclusion. We do have another study regarding HCW knowledge, attitudes, and practices toward TB infection control in Georgia that will soon be completed, and in that study we will evaluate the relationship between gender and compliance with TB infection control measures, including respirator use.

Such high rates of LTBI diagnostic test conversion emphasize the need for implementation of TB infection control measures. Our study provides valuable data on the rates of LTBI prevalence and LTBI test conversions in the country of Georgia. Prior to this study, there was no data on LTBI prevalence in HCWs from non-TB facilities in Georgia and no data on LTBI test conversions among HCWs from any healthcare facilities (TB and non-TB) in Georgia. In Georgia, patients with suspected TB are diagnosed and treated in specialized inpatient and outpatient TB facilities organized by the Georgian National Tuberculosis Program (NTP). Patients with TB generally do not receive care in other healthcare facilities; although persons with undiagnosed TB or suspected cases of TB may be seen at non-TB facilities and referred to a TB specialized facility later. TB infection control measures in Georgian healthcare facilities have been limited in the past, similar to most resource-limited countries. Like most low and middle-income countries, there are no routine programs in place in Georgia to screen healthcare workers for LTBI. The need for adequate TB infection control measures is further highlighted by HCWs' risk for being infected with MDR-TB. In 2010 among newly diagnosed TB cases in Georgia, 9.5% had MDR-TB, and among retreatment cases, 31% had MDR-TB [16]. There are no evidence-based LTBI treatment recommendations for M/XDR-TB contacts and no proven LTBI treatment regimens for M/XDR-TB contacts [29].

Our study had several limitations. Because of the high prevalence of LTBI, the number of uninfected HCWs who were at risk for LTBI test conversions was modest (77 were at risk for QFT-GIT conversion and 48 for TST conversion). HCWs are not routinely tested for LTBI in Georgia (and the vast majority of low and middle income countries) so there may have been selection or volunteer bias on HCWs who chose to participate in the study. Only 54% (154/285) of the HCWs enrolled in the study had repeated LTBI tests performed, which could have introduced bias with respect to conversion rates and risk factors. Finally, repeat testing occurred at different time intervals but this was clearly documented so we were able to calculate conversion rates over time.

Questions still remain regarding how an IGRA conversion should be defined and what its prognostic value is. Further evidence from IGRA serial testing studies, including long term follow up data of “converters” is needed, in order to be able to determine what changes in IGRA test values constitute the development of LTBI infection. Additionally, IGRA use for the serial testing of HCWs in low and middle-income countries is limited by the increased cost of the test over the TST and by the need for laboratory facilities and trained personnel to perform the IGRA test. In resource-limited, highly endemic TB countries, resources would likely be better spent on strengthening TB infection control measures than on the extra cost of IGRA screening. However, studies that show rates of LTBI test conversion can be powerful motivators for demonstrating the need for improved TB infection control. The National TB Program in Georgia is not planning to implement routine LTBI screening for HCWs, but the results of this study have reinforced the need for TB infection control measures in the country of Georgia, a high burden MDR-TB country, particularly for healthcare facilities specializing in TB care.
