# Correction: Prevalence and Incidence of Latent Tuberculosis Infection in Georgian Healthcare Workers

**DOI:** 10.1371/annotation/1191cfc8-6f0f-498f-aae2-5452884615b7

**Published:** 2013-06-27

**Authors:** Jennifer A. Whitaker, Veriko Mirtskhulava, Maia Kipiani, Drew A. Harris, Nino Tabagari, Russell R. Kempker, Henry M. Blumberg

The name of the first author was listed incorrectly. 

The correct name is: Whitaker JA. The correct citation is: Whitaker JA, Mirtskhulava V, Kipiani M, Harris DA, Tabagari N, et al. (2013) Prevalence and Incidence of Latent Tuberculosis Infection in Georgian Healthcare Workers. PLoS ONE 8(3): e58202. doi:10.1371/journal.pone.0058202. 

The correct abbreviation of the name in Contributions is: JAW. 

